# Cerebral Venous Thrombosis: Clinical, Radiological, Biological, and Etiological Characteristics of a French Prospective Cohort (FPCCVT)—Comparison With ISCVT Cohort

**DOI:** 10.3389/fneur.2021.753110

**Published:** 2021-11-08

**Authors:** Aude Triquenot Bagan, Isabelle Crassard, Ludovic Drouet, Marianne Barbieux-Guillot, Raphaël Marlu, Emmanuelle Robinet-Borgomino, Pierre-Emmanuel Morange, Valérie Wolff, Lelia Grunebaum, Frédéric Klapczynski, Elisabeth André-Kerneis, Fernando Pico, Brigitte Martin-Bastenaire, Emmanuel Ellie, Fanny Menard, François Rouanet, Geneviève Freyburger, Gaëlle Godenèche, Hong-An Allano, Thierry Moulin, Guillaume Mourey, Laurent Derex, Micheline Berruyer, Gwénaëlle Runavot, Catherine Trichet, Fausto Viader, Agnès Le Querrec, Thomas Tarek Husein, Sophie Cluet-Dennetiere, Francisco Macian-Montoro, Magali Donnard, Benoît Guillon, Catherine Ternisien, Mathieu Zuber, Sophie Laplanche, Philippe Tassan, Jean-Yves Peeltier, Sandrine Canaple, Bertrand Roussel, Nicolas Gaillard, Emilie Scavazza, Véronique Le Cam Duchez

**Affiliations:** ^1^Department of Neurology, CHU Rouen, Rouen, France; ^2^Department of Neurology, Lariboisière University Hospital, Paris, France; ^3^Department of Biological Hematology, Lariboisière University Hospital, Paris, France; ^4^Department of Neurology, Grenoble University Hospital, Grenoble, France; ^5^Department of Biological Hematology, Grenoble University Hospital, Grenoble, France; ^6^Department of Neurology, Marseille University Hospital, Marseille, France; ^7^Aix Marseille Univ, INSERM, INRAE, C2VN, Marseille, France; ^8^Stroke Unit, Strasbourg University Hospital, Strasbourg, France; ^9^Department of Biological Hematology, Strasbourg University Hospital, Strasbourg, France; ^10^Department of Neurology, Meaux Hospital, Meaux, France; ^11^Department of Biological Hematology, Meaux Hospital, Meaux, France; ^12^Department of Neurology Versailles Hospital, Versailles, France; ^13^Department of Biological Hematology, Versailles Hospital, Versailles, France; ^14^Department of Neurology, Bayonne Hospital, Bayonne, France; ^15^Department of Biological Hematology, Bayonne Hospital, Bayonne, France; ^16^Department of Neurology, Bordeaux University Hospital, Bordeaux, France; ^17^Department of Biological Hematology, Etablissement Français du Sang, Bordeaux, France; ^18^Department of Neurology, La Rochelle Hospital, La Rochelle, France; ^19^Department of Biological Hematology, La Rochelle Hospital, La Rochelle, France; ^20^Department of Neurology, Besançon University Hospital, Besançon, France; ^21^Department of Biological Hematology, Etablissement Français du Sang, Besançon, France; ^22^Department of Neurology, Lyon University Hospital, Lyon, France; ^23^Department of Biological Hematology, Lyon University Hospital, Lyon, France; ^24^Department of Neurology, Argenteuil Hospital, Argenteuil, France; ^25^Department of Biological Hematology, Argenteuil Hospital, Argenteuil, France; ^26^Department of Neurology, Caen University Hospital, Caen, France; ^27^Department of Biological Hematology, Caen University Hospital, Caen, France; ^28^Department of Neurology, Compiègne Hospital, Compiègne, France; ^29^Department of Biological Hematology, Compiègne Hospital, Compiègne, France; ^30^Department of Neurology, Limoges University Hospital, Limoges, France; ^31^Department of Biological Hematology, Limoges University Hospital, Limoges, France; ^32^Department of Neurology, Nantes University Hospital, Nantes, France; ^33^Department of Biological Hematology, Nantes Univeristy Hospital, Nantes, France; ^34^Department of Neurology, Saint Joseph Hospital, Paris, France; ^35^Department of Biological Hematology, Saint Joseph Hospital, Paris, France; ^36^Department of Neurology, Poissy-Saint-Germain Hospital, Poissy, France; ^37^Department of Biological Hematology, Poissy-Saint-Germain Hospital, Poissy, France; ^38^Department of Neurology, Amiens University Hospital, Amiens, France; ^39^Department of Biological Hematology, Amiens University Hospital, Amiens, France; ^40^Department of Neurology, Perpignan Hospital, Perpignan, France; ^41^Department of Biological Hematology, Perpignan Hospital, Perpignan, France; ^42^Normandie Univ, UNIROUEN, INSERM U1096, CHU de Rouen, Service d'Hématologie Biologique, Rouen, France

**Keywords:** cerebral veins, thrombosis, French cohort, prospective observational study, sinus

## Abstract

**Introduction:** Cerebral venous thrombosis (CVT) is a rare disease with highly variable clinical presentation and outcome. Etiological assessment may be negative. The clinical and radiological presentation and evolution can be highly variable. The mechanisms involved in this variability remain unknown.

**Objective:** The aim of this multicenter French study registered on ClinicalTrials.gov (NCT02013635) was therefore to prospectively recruit a cohort of patients with cerebral venous thrombosis (FPCCVT) in order to study thrombin generation and clot degradation, and to evaluate their influence on clinical radiological characteristics. The first part of the study was to compare our cohort with a reference cohort.

**Methods:** This prospective, multicenter, French study was conducted from July 2011 to September 2016. Consecutive patients (aged >15 years) referred to the stroke units of 21 French centers and who had a diagnosis of symptomatic CVT were included. All patients gave their written informed consent. The diagnosis of CVT had to be confirmed by imaging. Clinical, radiological, biological, and etiological characteristics were recorded at baseline, at acute phase, at 3 months and at last follow-up visit. Thrombophilia screening and the choice of treatment were performed by the attending physician. All data were compared with data from the International Study on CVT published by Ferro et al.

**Results:** Two hundred thirty-one patients were included: 117 (50.6%) had isolated intracranial hypertension, 96 (41.5%) had focal syndrome. During hospitalization, 229 (99.1%) patients received anticoagulant treatment. Median length of hospital stay was 10 days. Five patients died during hospitalization (2.2%). At 3 months, 216 patients (97.0%) had follow-up with neurological data based on an outpatient visit. The mean duration of antithrombotic treatment was 9 months, and the mean time to last follow-up was 10.5 months. At the end of follow-up, eight patients had died, and 26 patients were lost to follow-up. At least one risk factor was identified in 200 patients.

**Conclusions:** We demonstrated that the FPCCVT cohort had radiological, biological, and etiological characteristics similar to the historical ISCVT cohort. Nevertheless, the initial clinical presentation was less severe in our study probably due to an improvement in diagnostic methods between the two studies.

## Introduction

Cerebral venous thrombosis (CVT) is a rare disease, which is very different from ischemic or hemorrhagic stroke regarding affected populations, clinical characteristics, and prognosis (1, 2). Moreover, its main clinical characteristic is its great diversity. This diversity affects the mode of onset of the disease, which can be highly variable with an acute mode (<2 days), a sub-acute mode (between 2 and 30 days) or a chronic mode (more than 30 days) (3). Moreover, its clinical presentation is also highly variable, ranging from high isolated intracranial hypertension to diffuse encephalopathy and the presence of focal signs associated or not with a picture of cranial hypertension (4).

Imaging for the diagnosis of CVT shows variable images of both the extension of lesions and their evolution when controlled remotely.

The mechanisms that may explain this variability in the mode of onset, the clinical and radiological presentations, and the evolution of this disease are not well-explained.

The aim of this multicenter French research program was therefore to prospectively recruit a cohort of patients with cerebral venous thrombosis (FPCCVT). Then, we described FPCCVT and compared it with a historical cohort but which is a reference: the cohort from International Study on Cerebral Vein and Dural Sinus Thrombosis (ISCVT cohort) (5).

## Materials and Methods

This prospective, multicenter, French study was conducted from July 2011 to September 2016. Consecutive patients (aged >15 years) referred to the stroke units of 21 French centers and who had a diagnosis of symptomatic CVT were included. A scientific committee was appointed to monitor the study and to evaluate the interest of future biobank research.

All patients (or their parents or guardians) gave their written informed consent. The study protocol was approved by the local review committee on human research. This French cohort “FPCCVT” was recruited through a clinical research program (N° 2010/087/HP) funded by the French Ministry of Health and registered on ClinicalTrials.gov (NCT02013635: Thrombin Generation and Thrombus Degradation in Cerebral Venous Thrombosis: Clinical and Radiological Correlations).

### Clinical and Radiological Characteristics

The diagnosis of CVT had to be confirmed by cerebral tomodensitometry venography (CT Venography) and/or magnetic resonance imaging (MRI) combined with MR venography (MRV) and/or digital subtraction angiography (DSA) following established diagnostic criteria (6).

Patients' data, including demographics, medical history, date of onset of CVT, date of hospitalization, time to clinical and radiological confirmation of CVT, treatments and procedures, in-hospital mortality, and ambulatory status at discharge were recorded for each patient. Clinical status was evaluated at admission using Glasgow Coma Scale (GCS) score and the National Institutes of Health Stroke Scale (NIHSS). Presenting syndromes were classified as isolated intracranial hypertension and/or isolated headache, focal syndrome, cavernous sinus syndrome, and diffuse encephalopathy.

If not done at admission, cerebral MRI combined with MRV had to be performed within 72 h. Radiological evolution was evaluated at 3 months after inclusion using cerebral imaging, preferably cerebral MRI with MRV. “Partial recanalization” was defined as recanalization of at least one previously thrombosed sinus or vein in an individual patient with CVT. “No recanalization” was defined as total absence of any type of recanalization in any previously thrombosed sinus or vein. “Complete recanalization” was considered when all previously thrombosed structures met the criteria of complete recanalization.

### Etiological Characteristics, Treatment, and Follow-Up

A list of potential risk factors for CVT was attached to the inclusion form to assist investigators with etiological workup, but thrombophilia screening was performed by the attending physician.

The choice and the duration of anticoagulant treatment were at the discretion of the neurologist. All etiological assessments and treatments were systematically recorded during hospitalization, at discharge, and at each follow-up visit. Follow-up visits were performed at 3 months and 1 month after stopping antithrombotic treatment or after a maximum of 13 months if antithrombotic treatment was not stopped. At each follow-up visit, data recorded were current medications (antithrombotic, antiepileptic, etc.) and disability [according to modified Rankin Scale (mRS)]. Outcome was classified according to mRS as good functional outcome (mRS <2) or poor functional outcome (mRS ≥2).

### Statistical Analysis

Quantitative data were expressed as median and range or mean and standard deviation. Only qualitative data were compared between the two cohorts. These comparisons were performed with Chi^2^ test. *p*-values <0.05 were considered to be statistically significant.

## Results

### Clinical and Radiological Characteristics at Baseline

**Figure 1:**
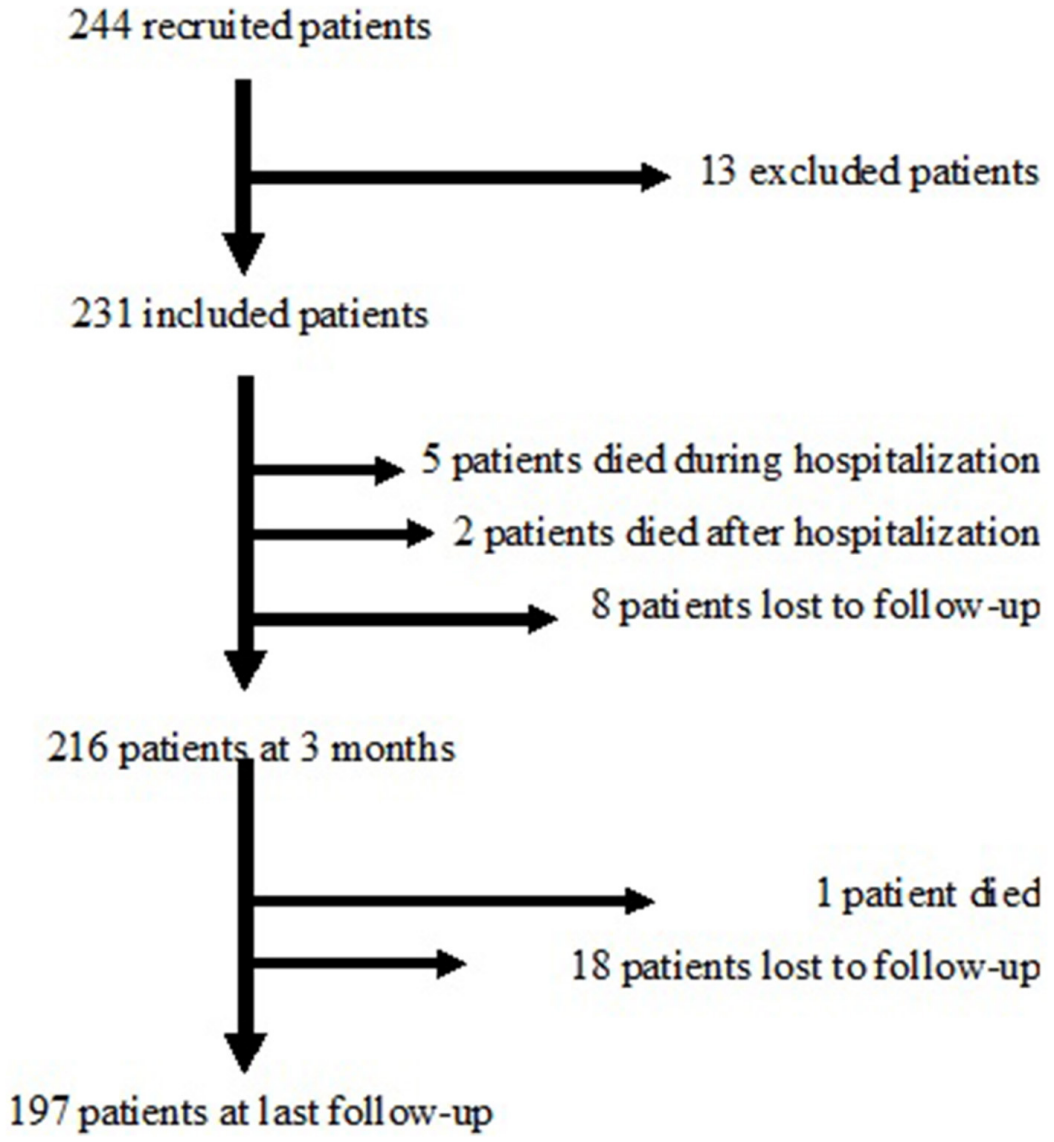
Flow chart of the study.

Two hundred forty-four patients were initially recruited, 13 of whom refused to participate or did not have a confirmed diagnosis, leaving 231 patients who were included in the FPCCVT. Figure 1 shows the flow chart of the study. Among them, the sex ratio (female/male) was 2.16 and the mean age of patients was 41.2 years (SD = 15.8).

Demographic and clinical characteristics at baseline are shown in Table 1. The median delay from onset of symptoms to diagnosis was 5 days (range 0–205). The mode of onset was acute in 35.5%, sub-acute in 56.7%, and chronic in 7.8% of patients.

**Table 1:** Demographic and clinical characteristics of the FPCCVT and ISCVT cohorts at baseline.

	**FPCCVT,** ***N*** **=** **231, 21 centers in France**		**ISCVT,** ***N*** **=** **624, 89 centers in 21 countries**		***p(X^2^)***
	**Number of cases**	**Missing**	**Number of cases**	**Missing**	
	**(% of results)**	**data (%)**	**(%)**	**data (%)**
Mean age (years)	41.2 ± 15.8	0	39.1	0	
Median age (years)	40 (range 16–85)		37 (range 16–86)		
Female	158 (68.4)	0	465 (74.5)	0	0.074
**Delay from onset of symptoms to hospitalization**					
Median	5		4		
Mean ± SD	11.35 ± 22.15		14.5 ± 57.4		
**Delay from onset of symptoms to diagnosis**					
Median	5		7		
Mean ± SD	11.8 ± 22.13		18.3 ± 59.4		
**Mode of onset of symptoms**					
Acute	82 (35.5)		232 (37.2)		
Subacute	131 (56.7)		346 (55.5)		0.89
Chronic	18 (7.8)		45 (7.2)		
**Symptoms and signs**					
Headache	215 (93.1)	0	553 (88.8)	1	0.8
Nausea and vomiting	116 (50.2)	0			
Papilledema	38 (16.5)	5 (2.2)	174 (28.3)	10	** <0.001**
Visual loss	26 (11.3)	0	82 (13.2)	3	0.44
Diplopia	18 (7.8)	0	84 (13.5)		**0.02**
Hemianopia	14 (6.0)	0			
Left or/and right paresis	57 (24.7)	0	232 (37.2)		** <0.001**
Left or/and right sensory	31 (13.4)	0	34 (5.4)		** <0.001**
**Symptoms**					
Aphasia	26 (11.3)	0	119 (19.1)		**0.007**
Focal seizure	15 (6.5)	0	122 (19.6)		** <0.001**
Seizure with	36 (15.5)		187 (30)		
**Generalization**					
Any seizure	51 (20.1)		245 (39.3)		** <0.001**
Mean GCS score	14.7	0			
Median GCS score	15 (range 8–15)				
GCS score <9	2 (0.9)		31 (5.2)		
GCS score between 9 and 13	11 (4.8)		83 (13.9)		** <0.001**
Mean NIHSS score ± SD	1.59 ± 3.75	0			
Median NIHSS score	0 (range 0–26)				
NIHSS = 0	149 (64.5)				
NIHSS <5	208 (90)				
NIHSS ≥5	23 (10)				

*NIHSS, National Institutes of Health Stroke Scale; GCS, Glasgow Coma Scale; SD, standard deviation; FPCCVT, French prospective cohort of patients with cerebral venous thrombosis; ISCVT, International Study on Cerebral Vein and Dural Sinus Thrombosis. Bold p values are significant p values*.

Thirty-six patients (15.7%) had a personal history of venous thrombosis, and 38/214 patients had a familial history of venous thromboembolic disease.

One hundred seventeen patients (50.6%) presented with isolated intracranial hypertension and/or isolated headache, 96 (41.5%) had focal syndrome, 16 (6.9%) had diffuse encephalopathy, 2 (0.9%) had cavernous sinus syndromes and 2 were comatose (GCS <9).

CVT diagnosis was made in emergency with CT Venography and/or with MRI and MRV as presented in Table 2. CVT diagnosis was made with DSA in only six patients with suspicion of CVT (2.6%). Thirty-two patients (13.9%) had one thrombosed vessel. The median number of sinuses with thrombosis was 3 per patient. The most frequently occluded vessels were the superior sagittal sinus in 111 patients (48%), the right transverse sinus in 110 patients (47.6%), the right sigmoid sinus in 93 patients (40.3%), the left transverse sinus in 78 patients (33.8%), and the left sigmoid sinus in 62 patients (26.8%). Seventeen patients (7.3%) presented with isolated cortical vein thrombosis and 17 patients (7.3%) with deep vein system thrombosis. Ninety-three (40.3%) patients had parenchymal cerebral lesions on MRI/CT (32 hemorrhagic lesion, 31 ischemic lesion, 27 both and 3 missing data).

**Table 2:** Radiological characteristics of the FPCCVT and ISCVT cohorts at baseline.

	**FPCCVT,** ***N*** **=** **231, 21 centers in France**		**ISCVT,** ***N*** **=** **624, 89 centers in 21 countries**		***p(X^2^)***
	**Number of cases**	**Missing**	**Number of cases**	**Missing**	
	**(% of results)**	**data (%)**	**(%)**	**data (%)**
**Diagnostic methods**					
CT venography	85 (36.8)		13 (2)		
MRI and MRV	71 (30.7)		443 (71)		
Several methods	69 (29.9)		89 (14)		
DSA	6 (2.6)		74 (12)		
**Thrombosed sinus/vein**					
Superior sagittal sinus	111 (48.3)		313 (62)		0.58
Lateral sinus, left	81 (35.1)		279 (44.7)		**0.01**
Lateral sinus, right	119 (51.5)		257 (41.2)		**0.007**
Straight sinus	31 (13.4)		112 (18)		0.12
Isolated cortical vein	17 (7.3)		107 (17.1)	1	** <0.001**
Deep vein system	17(7.3)		68 (10.9)	2	0.12
Cerebellar veins	4 (1.7)		3 (0.3)	2	0.07
Cavernous sinus	1 (0.4)		8 (0.4)	1	0.28
**Any parenchymal lesions**	93 (40.3)	3	392 (62.9)		** <0.001**
Ischemic	32 (13.9)		245 (39.3)	1	** <0.001**
Hemorrhagic	31 (13.4)		290 (46.5)	2	** <0.001**
Both	27 (11.7)				

*DSA, digital subtraction angiography; CT, cerebral tomodensitometry, MRI, magnetic resonance imaging; MRV, magnetic resonance venography. Bold p values are significant p values*.

### Acute Phase

The routine biological characteristics of the 231 patients are presented in Table 3. D-dimer assay was done locally in emergency in 161 patients (69.7%). According to the technique used in each center, values were above the exclusion threshold in 126 patients (78.3%). Among the 38 patients with anemia, 8 had a level of hemoglobin between 5 and 10 g/dl and 2 had hemoglobin below 5 g/dl. Moreover, among the 13 patients with thrombocytopenia, 2 had a platelet count below 100 G/L, and 1 had a platelet count lower than 30 G/L. During hospitalization, 229 (99.1%) patients received anticoagulant treatment (Table 4). The main anticoagulants used were unfractioned heparin and low molecular weight heparin.

**Table 3:** Routine biological characteristics of the 231 study patients.

	**Median [range]**,	**Missing data**
	**Number of patients**	**N (%)**
	**with abnormalities**	
Leucocytes (G/L)	9.2 [2–85.2]	1 (0.4)
Hemoglobin (g/dl)	13.7 [2.4–21.5]	1 (0.4)
Anemia (WHO definition)	38 (8M/30F)	1 (0.4)
Polycytemia (WHO definition)	12	
Platelets (G/L)	223 [27–1,361]	
Thrombocytopenia (<150 G/L)	13	
Thrombocytosis (>450 G/L)	8	
Ferritin (μg/L)	125 [3.8–1,379]	108 (46.8)
Decreased ferritin	11 (11F)	
Increased ferritin (>200 μg/L)	35 (7 without inflammation)	
Fibrinogen (g/L)	4 [0.5–7.6]	33 (14.3)
Fibrinogen >4 g/L	98	
C reactive protein (mg/L)	13 [0.5–194]	30 (13.0)
C reactive protein >5 mg/L	152 patients	
TSH (mUI/L)	1.39 [0.01–9.91]	46 (19.9)
Decreased TSH <0.5 mUI/L	20	
Increased TSH >5 mUI/L	8	
Hemoglobin A_1_c (%)	5.5 [3.3–10.9]	93 (40.3)
Hemoglobin A_1_c >6.5 %	10	
Creatinine (μmol/L)	66 [34–192]	2 (0.9)
Creatinine >110 μmol/L	5	
Bilirubin total (μmol/L)	9 [2–61]	23 (10.0)
Bilirubin > 17 μmol/L	19	
γGT (UI/L)	30.5 [7–716]	23 (10.0)
γGT >2N	27 (12M/25F)	
ASAT (UI/L)	20 [3–207]	16 (6.9)
ASAT >2N	3 (3M)	
ALAT (UI/L)	22 [6–95]	17 (7.4)
ALAT >2N	3 (3M)	
Total cholesterol (mmol/L)	4.9 [1.6–8.6]	45 (19.5)
Triglycerides (mmol/L)	1.36 [0.27–6.6]	44 (19.0)
Blood group		120 (51.9)
Group O	24	
Group A	62	
Group B	16	
Group AB	9	

*M, male; F, female; TSH, thyroid stimulating hormone; γGT, gamma glutamyl transferase; ASAT, aspartate amino-transferase; ALAT, alanine amino-transferase*.

**Table 4:** Treatments in the acute phase and outcomes at the end of follow-up in the FPCCVT and ISCVT cohorts.

	**FPCCVT**,	**ISCVT**,	***p(X^2^)***
	***N* = 231**,	***N* = 624**	
	**21 centers in France**	**89 centers in 21 countries**	
	**Number of cases (%)**	**Number of cases (%)**	
**Treatments in acute phase**			
Heparin			
UFH (IV + SC)	140 (61)	401 (64)	0.32
LMWH therapeutic dosage	84 (36)	218 (34.9)	0.98
LMWH prophylactic dosage	1 (0.4)	9 (1.4)	
Oral anticoagulant treatment			
VKA	1 (0.4)		
DOAC	4 (1.7)		
Antiplatelet drugs	5 (never alone)	37 (5.9)	
No anticoagulant treatment	2 (0.9)		
Other treatments			
External shunts	2 (0.9)	10 (1.6)	
Decompressive craniotomy	2 (0.9)	9 (1.4)	
Local thrombolysis	0	13 (2.1)	
Additional treatments			
Antalgic drugs	199 (86.1)		
Antiepileptics	59 (25.5)	277 (44.4)	** <0.001**
Osmotherapy	46 (19.9)	82 (13.2)	0.1
Steroids	19 (8.2)	150 (24.1)	** <0.001**
**At the end of follow-up:**	N = 205	N = 564	
	Median duration = 11.9 months	Median duration = 16 months	
Lost to follow-up	26 (11.3)	8 (1.3)	** <0.001**
Median duration of anticoagulant treatment	213 day	231 days	
Recurrent CVT	2 (1)	14 (2.2)	0.19
Death	8 (3.4)	52 (8.3)	**0.01**
Rankin scale score			
<2	181 (88.3)	493 (87.4)	0.46
≥2	24 (11.7)	79 (14)	

*UFH, unfractioned heparin; LMWH, low molecular weight heparin; VKA, vitamin K antagonists; DOAC, direct oral anticoagulant. Bold p values are significant p values*.

### At Discharge

At discharge, data were available for all 231 patients. Median length of hospital stay was 10 days (mean = 12.4, standard deviation = 7.6). Five patients (2.2%) died during hospitalization, four of brain herniation (despite decompressive craniectomy in one patient) and one of uncontrolled cancer.

Two hundred eight patients (90.0%) returned home, 16 (6.9%) were transferred to a rehabilitation center, and 2 patients were transferred to another medical facility. Two hundred twenty-three (98.7%) of the 226 surviving patients were prescribed curative anticoagulant therapy (188 oral VKA, 18 subcutaneous LMWH, 1 subcutaneous UFH, and 17 DOAC). Forty-six of the 51 patients who had epileptic manifestations were prescribed antiepileptics. Sixteen patients without a history of seizure were treated, and 10 among them presented with cerebral lesions on MRI.

### At 3 Months

At 3 months, 216 patients (97.0%) had follow-up with neurological data based on an outpatient visit. Two more patients died as a result of underlying conditions such as cancer and eight were lost to follow-up. For these eight patients lost to follow-up before the visit at 3 months, their condition on the day of hospital discharge was considered for the last follow-up visit. One hundred eighty patients (80.7%) had good functional outcome (mRS <2), and 36 (16.6%) had poor outcome (mRS ≥ 2). Two hundred thirteen patients (98.6%) received anticoagulant treatment, 46 (21.3%) antiepileptic drugs, and 20 (9.3%) anti-edematous treatment. Fifty-six (25.9%) patients were treated with antalgics. Cerebral venous imaging was performed in 211 patients (97.7%) after a median delay of 94.3 days (mean 92, SD: 21.4). Cerebral imaging was MRI with MRV in 202 cases and CT venography in 9 cases. Venous recanalization was achieved in 193 patients (91.4%), complete in 81 (38.4%), and partial in 112 (53.1%). Fifteen patients (7.1%) had no recanalization, and 4 patients (1.9%) presented new sinus or cortical vein thrombosis.

### Last Follow-Up

The main characteristics of the last follow-up are presented in Table 4. The mean and median delays to last follow-up were 10.5 and 11.9 months (range 76–543 months), respectively, for 197 (88.3%) of 223 surviving patients. One more patient died of cancer and 18 additional patients were lost to follow-up. For these 18 patients lost to follow-up after the visit at 3 months, their condition on the day of the visit at 3 months was considered for the last follow-up visit.

Among the 205 patients followed up, 97 (47.3%) were no longer treated with anticoagulants, 85 patients (41.5%) were still on anticoagulants, and 20 (9.8%) were on aspirin.

One hundred eighty-one patients had good functional outcome (mRS <2), and 24 had poor outcome (mRS ≥ 2).

### Etiological Characteristics

Risk factors and etiologies of CVT are presented in Table 5. At least one risk factor was identified in 200 of the 231 patients (86.6%). Some risk factors were known before the cerebral thrombotic event: six factor V Leiden mutations, one factor II 20210A mutation, one antithrombin deficiency, and one myeloproliferative disease. Others were discovered at the time of the cerebral event. Oral estrogen and progestin treatment users comprised the largest group (91 patients, 57.6%, all women). Among these 91 patients, 11 had an association of oral contraceptive and tobacco; 13.9% (*n* = 32) of all patients were regular tobacco smokers. Eighty-eight women were on oral estrogen and progestin contraceptives and the median delay between the beginning of treatment and thrombosis was 36 months (1–372). One woman was pregnant, and four had recently given birth. Systemic diseases were reported in 48 patients (20.8%). A local condition was identified in 31 patients (13.4%). The most frequent was intracranial hypotension (10 cases), spontaneous or after lumbar puncture. Local infection was associated in nine patients. Two hundred twenty-eight patients (98.7%) were tested for biological thrombophilia with coagulation inhibitor assays, Factor V Leiden and prothrombin G20210A mutations, and antiphospholipid syndrome. Nevertheless, most of the time, the thrombophilia assessment was incomplete, and for antiphospholipid syndrome, in particular, only 106 patients had a full search with lupus anticoagulant, anti-cardiolipin, and anti-beta2GPI antibodies assays. Regarding genetic thrombophilia, five patients had more than one thrombophilia: one antithrombin deficiency and factor II 20210A mutation, one protein S deficiency and Factor V Leiden mutation, two protein S deficiencies and Factor II 20210A mutation, and one association of homozygous factor V Leiden mutation and heterozygous factor II 20210A mutation. The factor II 20210A mutation was more frequent than the factor V Leiden mutation. In women, these mutations were often associated with oral estrogen contraceptive use, respectively, 10 associations with factor II 2010A and 6 associations with factor V Leiden mutation.

**Table 5:** Etiological characteristics of the FPCCVT and ISCVT cohort.

	**FPCCVT,** ***N*** **=** **231, 21 centers in France**		**ISCVT,** ***N*** **=** **624, 89 centers in 21 countries**		***p(X^2^)***
	**Number of cases**	**Missing**	**Number of cases**	**Missing**	
	**(% of results)**	**data (%)**	**(%)**	**data (%)**
None identified	31 (15.5)		78 (12.5)		0.72
**Thrombophilia**			213 (34.1)		
**Genetic**	51 (22.1)		140 (22.4)		0.91
Antithrombin deficiency	3 (1.4)	20			
Protein C deficiency	4 (1.9)	15			
Protein S deficiency	10 (4.7)	16			
Activated Protein C resistance (APCR)	14 (12.7)	121			
Factor V Leiden mutation	62 (27.2)	65^*^			
Factor II 20210A mutation	22 (9.5)	40			
**Acquired**	62 (27.2)		98 (15.7)		** <0.001**
**Antiphospholipid AB**	22 (9.5)		40 (5.9)		0.12
Anticardiolipin AB	1 IgG (0.5)	21			
Anti β2GPI AB	0	13			
Lupus Anticoagulant	21 (7.4)	92			
**Hyperhomocysteinemia**	41 (23)	53	28 (4.5)		** <0.001**
**Systemic diseases**	48 (20.8)				
Myeloproliferative neoplasm	4 (1.7)		18 (2.9)		0.34
Extra cerebral neoplasia	9 (3.9)		20 (3.2)		0.62
Thrombotic thrombocytopenic purpura	7				
Inflammatory bowel disease	7 (3)		10 (1.6)		0.18
Anemia	38 (16.4)		58 (9.2)	Not precise	**0.003**
Behçet's disease	2 (0.9)		6 (1)		0.9
Inflammatory arthritis	3 (1.3)		1 (0.2)		**0.03**
Thyroid disease	19 (8.2)		11 (1.7)		** <0.001**
SLE	1 (0.5)		7 (1)		0.35
**“Female-related risk factors”**					
Oral contraceptives (% of women ≤ 50 years)	88 (71)		207 (54.3)		**0.001**
Hormonal replacement therapy (% of women >50 years)	3 (8.8)		27 (32.1)		**0.008**
Pregnancy/puerperium (% of women ≤ 50 years)	5 (3.2)		77 (20.2)		** <0.001**
**Local conditions**	31 (13.4)		104 (16.7)		0.25
Local infection and trauma	11		71		
Low CSF pressure	10				
Cerebral tumor	4		14		
Arteriovenous malformation	3		1		
Local neurosurgery	3				
Local tumor	3		4 (0.6)		
Cerebral hemorrhage	1				

*SLE, systemic lupus erythematosus; CSF, cerebrospinal fluid*.

* *Among the 65 factor V Leiden mutations not searched: 46 had normal APCR. Bold p values are significant p values*.

### Comparison Between French Prospective Cohort of Cerebral Venous Thrombosis and International Study on Cerebral Vein and Dural Sinus Thrombosis

The two cohorts were similar regarding the mean age of patients, the sex ratio, the delay from onset of symptoms to hospitalization, and the mode of onset of symptoms. Nevertheless, the delay from onset of symptoms to hospitalization or diagnosis was slightly shorter in our study than in the ISCVT study.

In the ISCVT cohort, 143 patients (22.9%) presented with isolated intracranial hypertension (vs. 41.5% in FPCCVT). The initial clinical presentation was slightly different between the two cohorts, with more seizures, visual symptoms, and different focal symptoms in the ISCVT cohort. Moreover, more patients were comatose in the ISCVT cohort. The initial clinical presentation was less severe in our cohort.

The initial anticoagulant treatment was similar between the two cohorts. In FPCCVT, fewer antiepileptic drugs were prescribed because of fewer seizures at initial clinical presentation (22 vs. 50% in ISCVT). In the ISCVT cohort, the median hospital stay was longer: 17 days (mean = 20.4, SD = 14.3), and 27 patients died during hospitalization (4.3%) without a significant difference (*p* = 0.13). At the end of follow-up, even if the median duration of follow-up was longer in ISCVT than in FPCCVT, outcomes, recurrences, and deaths were not significantly different. Nevertheless, there were more patients lost to follow-up in FPCCVT.

Concerning etiologies, the number of oral contraceptive users was not significantly different between the two cohorts (*p* = 0.11). In the ISCVT cohort, there were significantly more women with pregnancy/puerperium.

The two cohorts were not significantly different either for patients without risk factors, or for hereditary thrombophilia. Concerning acquired thrombophilia, there was a significant difference for the number of patients with hyperhomocysteinemia, but in our study, the threshold was higher than 15 mmol/L but was not known in the ISCVT study.

## Discussion

In this prospective multicenter French study, we evaluated the clinical, radiological, biological, and etiological characteristics of a large and representative cohort of patients with cerebral venous thrombosis. Next, we compared our cohort with the ISCVT cohort, which is the largest published prospective cohort. Since 2004, other cohorts have been published but most are retrospective cohorts.

The two cohorts were similar in mean patient age and sex ratio. The mean time from symptom onset to diagnosis was somewhat shorter in our cohort than in the ISCVT. This may explain why the initial clinical presentation was less severe.

This less severe clinical presentation is reflected in more isolated intracranial hypertension, fewer seizures, and fewer patients with a Glasgow score <13 and a shorter hospital stay. These differences may be explained by the fact that ISCVT patients were included between 1998 and 2001 and FPCCVT patients between 2011 and 2016. Coutinho et al. previously showed an improvement over time in initial CVT presentations due to improved diagnostic methods (7). Twenty-one percent of our patients had epileptic manifestations at the acute phase of CVT, which is less than in the ISCVT cohort but consistent with data in the literature for severe CVT and peripartum CVT (8). Although the initial presentation was less severe in FPCCVT, the subsequent evolution was not so different. At the end of follow-up, there was the same proportion of recurrences and the same proportion of patients with good functional evolution on mRS. However, there was less mortality. This lower mortality cannot be related to a difference in anticoagulant treatment. In fact, unfractionated heparin and low molecular weight heparin were the treatments most commonly used in the two cohorts. All but two of our patients were treated with anticoagulants in the acute phase of CVT despite a relatively high rate of ischemic and/or hemorrhagic brain lesions. This result is in line with recent European recommendations of good practices (9). The mean duration of antithrombotic treatment was 9 months in this series. At the end of the study, almost one half of our patients were still on anticoagulants, which corresponds, according to the guidelines, to idiopathic CVT and to patients presenting with severe thrombophilia or permanent thrombotic risk factors such as myeloproliferative syndrome or cancer (9). Also, the mortality rate in our study was extremely low and was comparable with that in the literature. Coutinho et al., in a systematic review, reported a mortality rate of <5% in the most recent studies (7). The fact that study patients were hospitalized in neurology departments probably explains the non-inclusion of patients with the most severe presentation (severe consciousness alteration) requiring intensive care and/or neurosurgery upon admission.

Recurrences were rare, lower than the rate observed in the literature, but the follow-up in this study was short, on average 10.5 months (13 months maximum) (1), and patients were mainly on anticoagulants throughout follow-up.

The radiological characteristics of our patients at baseline were similar to those in the literature for localization of thrombosis and for parenchymal lesions. In the multicenter retrospective VENOST study (10), as in other studies (11, 12), the superior sagittal sinus was the most frequently occluded sinus, and ~60% of patients had no parenchymal lesion, as in our study but more than in the ISCVT cohort (38%). In contrast, in the VENOST study (10), 48.2% of patients had only one thrombosed vessel and only 13.8% in FPCCVT. We did not perform central review of imaging which could be considered as a methodological limitation. Our rate of complete recanalization was lower than that observed in the literature but we performed control MRI on average 94 days after inclusion (13). This shorter delay could explain the higher number of partial recanalizations observed in our study. However, the recanalization rate is in line with that of previous studies at 3–6 months for complete and partial recanalization (13–15).

Our study highlights heterogeneous etiological assessments (especially for thrombophilia screening) according to centers. However, inclusions finished in 2015, before the publication in 2017 of The European Stroke Organization guidelines (9). It would be interesting to reassess practices following this publication. Despite this heterogeneous assessment, only 13.4% of our patients presented CVT without a condition or risk factor, which is close to the lowest figures reported in the literature (1, 9). Moreover, etiological characteristics of our population were not really different from the ISCVT cohort particularly concerning inherited thrombophilia. Prevalence of inherited thrombophilia was similar in both the cohorts and in the VENOST study (10). In our study, factor II 20210A mutation was more frequent than factor V Leiden mutation as previously described (16, 17). In another study (18), factor V Leiden mutation was more frequent than factor II 20210A mutation. These discrepancies may be explained by the country where the studies were conducted because the prevalence of these two mutations was different according to the countries (19). In our cohort, factor II 20210A mutation was often associated with oral estrogen and progestin contraceptives as previously described (16). These results were in agreement with the fact that the association between factor V or factor II mutations and oral contraceptive use increased the risk of CVT (20, 21).

Concerning acquired thrombophilia, prevalences of antiphospholipid antibodies and hyperhomocysteinemia were different between the two cohorts. Several explanations are possible: first, the detection of these two risk factors was not done in all the patients of our cohort; second the definition of hyperhomocysteinemia was not given in the ISCVT study.

In addition, the related female risk factors were highly different between the two cohorts. Oral contraceptive use was much more common in our cohort. Our cohort was recruited only in France, while ISCVT was an international cohort including patients from European and non-European countries (Mexico). One possible explanation for this difference is that access to hormonal treatments was more difficult 10 years ago than today, especially in some countries such as Mexico where the proportion of women under 40 years of age on oral contraceptives was 15.6% in 2012 (22). Several retrospective cohorts described different percentages: 12% of women in Pakistan and the Middle East (11), 23.5% of women in Tunisia (12), and 13.9% in Turkey (10, 22). Moreover, some studies specified the percentage of women under 50 years old (5, 12, 22), while others did not (10, 11). There were also significantly fewer pregnant or post-partum women in our study than in others, where the proportion ranged from 20 to 54%. This discrepancy could be partially explained by the fact that puerperium is a more frequent risk factor in low-income countries (21, 23).

Anemia was more frequent in FPCCVT than in ISCVT but less frequent than in Tunisian population (28.5%) (12) or in the case-control study of Coutinho et al. (27%) (24). This discrepancy might be explained by the fact that the prevalence of anemia was not a specific research objective in ISCVT and that measurement of hemoglobin was not mandatory. This may have led to under-reporting of anemia, especially of milder cases. The prevalence of anemia in our study was similar to the results of a large registry from India in which hemoglobin concentrations were routinely measured and which reported anemia in 18.4% of patients; the authors used a similar definition for anemia as we did (25). Coutinho et al. (24) and Green et al. (26) concluded that anemia was a risk factor for CVT. In the same manner, thyroid diseases were significantly more frequent in FPCCVT than in ISCVT. Nevertheless, as anemia, the prevalence of thyroid disease was not a specific research objective in ISCVT, and the definition of thyroid disease was not given. Moreover, there are very few published studies on the involvement of thyroid disease in CVT and only case reports of hyperthyroidism (27, 28).

## Conclusion

In this first part of the study, we have demonstrated that the FPCCVT cohort had similar clinical, radiological, biological, and etiological characteristics to the historical ISCVT cohort. Only initial clinical presentation was less severe in FPCCVT, probably due to improvements in the diagnostic methods of CVT between the two studies.

## Data Availability Statement

The raw data supporting the conclusions of this article will be made available by the authors, without undue reservation.

## Ethics Statement

The studies involving human participants were reviewed and approved by Comité de Protection des Personnes Nord-Ouest 1, approval number CPP 2010-023. All patients (or their parents/legal guardians) gave their written informed consent to participate in this study.

## Author Contributions

ATB and VLCD are the principal investigators of this research program, drafted and revised the manuscript for important intellectual content, and are accountable for all aspects of the work. All other authors are investigators from the different centers. They are listed in order of the number of inclusions and participated in the proofreading of the manuscript. All authors contributed to the article and approved the submitted version.

## Funding

This work was supported by the Research program (No. 2010/087/HP) funded by the French Ministry of Health.

## Conflict of Interest

The authors declare that the research was conducted in the absence of any commercial or financial relationships that could be construed as a potential conflict of interest.

## Publisher's Note

All claims expressed in this article are solely those of the authors and do not necessarily represent those of their affiliated organizations, or those of the publisher, the editors and the reviewers. Any product that may be evaluated in this article, or claim that may be made by its manufacturer, is not guaranteed or endorsed by the publisher.

## References

[1] Dentali F Poli D Scoditti U Di Minno MN De Stefano V Siragusa S. Long-term outcomes of patients with cerebral vein thrombosis: a multicenter study. J Thromb Haemost. (2012) 10:1297–302. 10.1111/j.1538-7836.2012.04774.x 22578023

[2] Einhaupl K Bousser MG de Bruijn SF Ferro JM Martinelli I Masuhr F. EFNS guideline on the treatment of cerebral venous and sinus thrombosis. Eur J Neurol. (2006) 13:553–9. 10.1111/j.1468-1331.2006.01398.x 16796579

[3] Bousser MG. Cerebral venous thrombosis: diagnosis and management. J Neurol. (2000) 247:252–8. 10.1007/s004150050579 10836615

[4] Cumurciuc R Crassard I Sarov M Valade D Bousser MG. Headache as the only neurological sign of cerebral venous thrombosis: a series of 17 cases. J Neurol Neurosurg Psychiatry. (2005) 76:1084–7. 10.1136/jnnp.2004.056275 16024884 PMC1739763

[5] Ferro JM Canhao P Stam J Bousser MG Barinagarrementeria F. Prognosis of cerebral vein and dural sinus thrombosis: results of the International Study on Cerebral Vein and Dural Sinus Thrombosis (ISCVT). Stroke. (2004) 35:664–70. 10.1161/01.STR.0000117571.76197.26 14976332

[6] Bousser MG. RRR. Cerebral venous thrombosis. In: Warlow CP, Van Gijn J, editors. Major Problems in Neurology. London: WB Saunders (1997). p. 27–9. 10.1016/B978-012743170-3.50107-2

[7] Coutinho JM Zuurbier SM Stam J. Declining mortality in cerebral venous thrombosis: a systematic review. Stroke. (2014) 45:1338–41. 10.1161/STROKEAHA.113.004666 24699058

[8] Mehvari Habibabadi J Saadatnia M Tabrizi N. Seizure in cerebral venous and sinus thrombosis. Epilepsia Open. (2018) 3:316–22. 10.1002/epi4.12229 30187001 PMC6119760

[9] Ferro JM Bousser MG Canhao P Coutinho JM Crassard I Dentali F. European Stroke Organization guideline for the diagnosis and treatment of cerebral venous thrombosis - Endorsed by the European Academy of Neurology. Eur Stroke J. (2017) 2:195–221. 10.1177/2396987317719364 31008314 PMC6454824

[10] Duman T Uluduz D Midi I Bektas H Kablan Y Goksel BK. A multicenter study of 1144 patients with cerebral venous thrombosis: the VENOST study. J Stroke Cerebrovasc Dis. (2017) 26:1848–57. 10.1016/j.jstrokecerebrovasdis.2017.04.020 28583818

[11] Khealani BA Wasay M Saadah M Sultana E Mustafa S Khan FS. Cerebral venous thrombosis: a descriptive multicenter study of patients in Pakistan and Middle East. Stroke. (2008) 39:2707–11. 10.1161/STROKEAHA.107.512814 18635853

[12] Sassi SB Touati N Baccouche H Drissi C Romdhane NB Hentati F. Cerebral venous thrombosis: a Tunisian monocenter study on 160 patients. Clin Appl Thromb Hemost. (2017) 23:1005–9. 10.1177/1076029616665168 27582021

[13] Aguiar de Sousa D Lucas Neto L Canhao P Ferro JM. Recanalization in cerebral venous thrombosis. Stroke. (2018) 49:1828–35. 10.1161/STROKEAHA.118.022129 30021808

[14] Herweh C Griebe M Geisbusch C Szabo K Neumaier-Probst E Hennerici MG. Frequency and temporal profile of recanalization after cerebral vein and sinus thrombosis. Eur J Neurol. (2016) 23:681–7. 10.1111/ene.12901 26667584

[15] Putaala J Hiltunen S Salonen O Kaste M Tatlisumak T. Recanalization and its correlation to outcome after cerebral venous thrombosis. J Neurol Sci. (2010) 292:11–5. 10.1016/j.jns.2010.02.017 20206363

[16] Le Cam-Duchez V Bagan-Triquenot A Menard JF Mihout B Borg JY. Association of the protein C promoter CG haplotype and the factor II G20210A mutation is a risk factor for cerebral venous thrombosis. Blood Coagul Fibrinolysis. (2005) 16:495–500. 10.1097/01.mbc.0000184738.27723.b2 16175009

[17] Marjot T Yadav S Hasan N Bentley P Sharma P. Genes associated with adult cerebral venous thrombosis. Stroke. (2011) 42:913–8. 10.1161/STROKEAHA.110.602672 21350198

[18] Beye A Pindur G. Clinical significance of factor V Leiden and prothrombin G20210A-mutations in cerebral venous thrombosis - comparison with arterial ischemic stroke. Clin Hemorheol Microcirc. (2017) 67:261–6. 10.3233/CH-179207 28869458

[19] Zivelin A Griffin JH Xu X Pabinger I Samama M Conard J. A single genetic origin for a common Caucasian risk factor for venous thrombosis. Blood. (1997) 89:397–402. 10.1182/blood.V89.2.397 9002940

[20] Saadatnia M Tajmirriahi M. Hormonal contraceptives as a risk factor for cerebral venous and sinus thrombosis. Acta Neurol Scand. (2007) 115:295–300. 10.1111/j.1600-0404.2007.00824.x 17489938

[21] Martinelli I. Cerebral vein thrombosis. Thromb Res. (2013) 131(Suppl. 1):S51–4. 10.1016/S0049-3848(13)70022-7 23452743

[22] Ruiz-Sandoval JL Chiquete E Banuelos-Becerra LJ Torres-Anguiano C Gonzalez-Padilla C Arauz A. Cerebral venous thrombosis in a Mexican multicenter registry of acute cerebrovascular disease: the RENAMEVASC study. J Stroke Cerebrovasc Dis. (2012) 21:395–400. 10.1016/j.jstrokecerebrovasdis.2011.01.001 21367622

[23] Ferro JM Canhao P Aguiar de Sousa D. Cerebral venous thrombosis. Presse Med. (2016) 45(12 Pt 2):e429–50. 10.1016/j.lpm.2016.10.007 27816347

[24] Coutinho JM Zuurbier SM Gaartman AE Dikstaal AA Stam J Middeldorp S. Association between anemia and cerebral venous thrombosis: case-control study. Stroke. (2015) 46:2735–40. 10.1161/STROKEAHA.115.009843 26272383

[25] Narayan D Kaul S Ravishankar K Suryaprabha T Bandaru VC Mridula KR. Risk factors, clinical profile, and long-term outcome of 428 patients of cerebral sinus venous thrombosis: insights from Nizam's Institute Venous Stroke Registry, Hyderabad (India). Neurol India. (2012) 60:154–9. 10.4103/0028-3886.96388 22626695

[26] Green M Styles T Russell T Sada C Jallow E Stewart J. Non-genetic and genetic risk factors for adult cerebral venous thrombosis. Thromb Res. (2018) 169:15–22. 10.1016/j.thromres.2018.07.005 30005273

[27] Liu JC Huang HY Hsu YT. Hyperthyroidism and thrombophilia in cerebral arterial and venous thrombosis: a case report and critical review. Neurologist. (2015) 19:53–5. 10.1097/NRL.0000000000000005 25607334

[28] Mouton S Nighoghossian N Berruyer M Derex L Philippeau F Cakmak S. Hyperthyroidism and cerebral venous thrombosis. Eur Neurol. (2005) 54:78–80. 10.1159/000087717 16118502

